# Characterization of probiotic properties and development of banana powder enriched with freeze-dried *Lacticaseibacillus paracasei* probiotics

**DOI:** 10.1016/j.heliyon.2022.e11063

**Published:** 2022-10-12

**Authors:** Phoomjai Sornsenee, Siriphorn Chimplee, Phanvasri Saengsuwan, Chonticha Romyasamit

**Affiliations:** a Department of Family and Preventive Medicine, Faculty of Medicine, Prince of Songkla University, Songkhla 90110, Thailand; b Department of Biomedical Sciences and Biomedical Engineering, Faculty of Medicine, Prince of Songkla University, Hat Yai, Songkhla, 90110, Thailand; c Department of Medical Technology, School of Allied Health Sciences, Walailak University, Nakhon Si Thammarat 80160, Thailand; d Research Center of Excellence in Innovation of Essential Oil, Walailak University, Nakhon Si Thammarat, 80160, Thailand

**Keywords:** *Lacticaseibacillus paracasei*, Probiotic, Functional food, Freeze drying, Banana

## Abstract

*Lacticaseibacillus paracasei* is one of the probiotic bacteria widely identified from fermented foods. The application of *L. paracasei* is commonly used in dairy and non-dairy products. To investigate the probiotic properties of *L. paracasei* cells including their acid, pepsin, pancreatin, and bile salt tolerances; adhesion ability; antipathogen activity; and antibiotic susceptibility, *L. paracasei* cells were incorporated into skim milk and lyophilized by freeze drying. Freeze-dried probiotic cells were add to green banana powder and low moisture additive food matrices and a storage analysis of the product was performed. The result showed that *L. paracasei* cells possessed potentially beneficial probiotic properties to survive stress in the gastrointestinal tract (GIT) and functional abilities as an anti-enteropathogenic agent; they were also safe to use and displayed antibiotic properties. Furthermore, the probiotic freeze-drying technique preserved high probiotic cell survivability (10^11^ CFU/g). In term of prolonged storage (60 days), the powder product was stable and maintained probiotic survival (10^7^ CFU/g) while excluding non-probiotic growth. In conclusion, *L. paracasei* displayed probiotic properties in the GIT and was judged to be a highly acceptable product as a probiotics–banana rehydrated beverage.

## 1 Introduction

Probiotics are defined as “*live microorganisms which, when administered in adequate amounts, confer a health benefit on the host*” [1, 2]. *Lacticaseibacillus* belongs to the largest lactic acid bacteria in *Lactobacillaceae* family and includes various facultative anaerobic species of rod-shaped Gram-positive bacteria [3, 4]. Apart from the genus *Bifidobacterium, Lacticaseibacillus* (previous name is *Lactobacill*us) is the most widely used genus in probiotic food production—particularly in fermented dairy products (milk, yogurt, bio-yogurt, Kefir, and cheese) [5, 6]. Recently, applications of *Lacticaseibacillus* species have also been developed for non-diary fermented probiotic products in soy milk, fruits, vegetables, vegetable juices, meat, and bread [5, 7].

*Lacticaseibacillus paracasei* is a member of potentially probiotic bacteria related to the *L. casei* group (*L. casei* and *L. rhamnosus*) [8]. *L. paracasei* are normal microbiota in the mammalian gut [9, 10], and are identified from many fermented foods [11, 12], and are used extensively in starter cultures for dairy products, often to improve flavor and texture of products, and for their health-related advantages [8, 13]. Mantzourani et al. isolated potential probiotic *L. paracasei* cells from Kefir grains, and applied the probiotics to feta-type cheese production [14]. However, the study of *L. paracasei* application to create novel probiotic products remains under-researched.

Species of *Lacticaseibacillus* probiotics are widely marketed for human consumption in several available preparations as liquid, capsule, and powder products [15, 16]. The global probiotics market size was estimated at $54.77 billion in 2020 and is expected to increase to $95.25 billion by 2028 [17]. Furthermore, dry probiotic products are projected to be a key area in market segmentation generating millions of USD in United States revenue between 2017 and 2028 [17]. The probiotic powder recipe could be freely incorporated with dietary supplements or other “dry” food matrices. Dried probiotics products are also more suited to consumption and handling due to their reduced volume; the reduced weight of the product helps to lower the packaging, transport, and storage costs [18, 19].

Among the various drying techniques, freeze drying (FD) or lyophilization has become one of the most important processes for preservation of food products [18]. FD is widely used to produce probiotic cells in powder form and preserves cell viability [20]. FD also provides better food quality because the temperatures used during the whole process remain low. At low temperatures of lyophilization, the color, aroma, taste, texture, and nutrients in the product remain intact at a higher recovery rate [18, 21]. Cryoprotectant medium incorporated with FD helps to protect probiotic cells, leading to increased product quality and extended shelf-life [18]. Skim milk protein (SMP) is a nonpermeating cryoprotective compound enhancing bacteria preservation [22, 23]. After lyophilization, the optimum concentration of SMP (10% w/v) effectively preserved probiotics viability (>90%) in *Enterococcus faecalis* and *Lacticaseibacillus* species such as *L. fermentum*, *L. rhamnosus*, *L. casei*, and *L. paracasei* [24, 25]. In long-term storage, 10% SMP maintained 70% survival of *L. fermentum* at the maximum (1 year) analysis [25].

The banana, belonging to the family *Musaceae*, is a common plant in tropical and subtropical countries [26]. Banana contains water (75%) and carbohydrate (25%). Trace amount of protein, fat, minerals (K, Mg, P, Fe, and Ca), and vitamins (provitamins A, B, and C) were also detected in the banana [27, 28]. In the green stage, bananas comprise 60%–80% of carbohydrates indigestible by humans—such as fibers (cellulose, hemicelluloses, and lignin) and starch/resistant starch content [29, 30]. The indigestible carbohydrates of banana represent a prebiotic property as a source of probiotic bacterial growth (of, for instance, *Lactobacilli* spp.) and in the production of short-chain fatty acids during probiotic fermentation [31, 32]. Therefore, bananas are a potential carbohydrate source for probiotic food supplementation. In a previous study, Powthong et al. investigated the potential prebiotic effects of powders obtained from bananas. The result showed that bananas have prebiotic properties such as the ability to hold water and oil, antioxidant content, and the ability to stimulate lactobacilli growth. As a result, bananas could be a good source of prebiotics [26]. To the best of our knowledge, there are no studies that have evaluated the effect of banana powder enriched with *L. paracasei* T0901 probiotic.

Here, aimed to investigate the properties of *L. paracasei* cells as probiotics. It incorporated the application of freeze-dried *L. paracasei* probiotic cells with green banana powder and products with lower water activity (a_w_ < 0.6) and moisture (<25%)—such as granulated sugar, cocoa powder, non-dairy creamer, and skimmed milk [33, 34]. This study aimed to develop the product as a probiotic powder beverage. The banana and granulated sugar derived from the nipa palm plant are also low-cost and easy to handle throughout the agricultural region of southern Thailand, and richly prebiotic sources for probiotics [26, 35, 36, 37].

## 2 Materials and methods

### 2.1 Probiotic microorganism

The *L. paracasei* strain T0901 was previously isolated from fermented palm sap and identified by Sornsenee et al. [11]. Briefly, *L. paracasei* T0901 was identified according to Bergey's manual classification and confirmed by matrix-assisted laser desorption ionization-time of flight (MALDI-TOF) typing. Amplification of 16S rRNA and sequencing were also performed. *L. paracasei* was used as a probiotic in further experiments in this study.

### 2.2 In vitro tests simulating the human GIT

#### 2.2.1 Characterization of probiotic properties

##### 2.2.1.1 Acid tolerance

The acid tolerances (pH 2.0 and 3.0) of probiotics were examined according to Sornsenee et al. [11]. Briefly, *L. paracasei* cells were harvested by centrifugation. After washing the pellet, the cell suspension was treated with hydrochloric acid (HCl) to pH 2.0 or 3.0. After incubating the cell suspension at 37 °C for 3 h, and enumerated the acid tolerance of the *L. paracasei* cells by cell viability on MRS agar plates, the bacterial survival rate (%) was calculated using the following equation (1):

(1) Survival rate (%) = (Final (Log CFU/mL)/Initial (Log CFU/mL)) × 100

##### 2.2.1.2 Pepsin and pancreatin tolerances

The digested tolerances of pepsin and pancreatin were investigated according to Sornsenee et al. [11]. In brief, 3 g/L of pepsin and 1 g/L of pancreatin (Sigma-Aldrich) were prepared in MRS broth with pH 2.0 and pH 8.0 adjustments, respectively. Subsequently, the overnight bacterial culture was harvested. The washed the pellet was then resuspended in MRS broth containing indicated pepsin or pancreatin solutions. Cell suspensions incubated at 37 °C for 3 h (for pepsin) or 4 h (for pancreatin), and counted the *L. paracasei* colonies on the MRS plates. The percentage survival rate was calculated using Eq. (1).

##### 2.2.1.3 Bile salt tolerance

The bile tolerance determined according to Sornsenee et al. [11]. Briefly cells were harvested from an overnight culture of *L. paracasei* by centrifugation and the bacterial cells were adjusted to 0.3% (w/v) bile salt (Sigma-Aldrich) in MRS broth and then incubated them at 37 °C for 4 h. The bacterial viability was enumerated in toleration to the bile on an MRS agar plate. The percentage survival rate was calculated using Eq. (1).

##### 2.2.1.4 Cell surface hydrophobicity

Hydrophobicity of *Lacticaseibacillus* spp. was determined using xylene extraction [11]. Briefly, the cells from an overnight culture of *L. paracasei* were harvested by centrifugation. After washing the pellet, it was resuspended in phosphate-buffered saline and the absorbance was measured at OD_600_ nm. Subsequently, xylene was added to the cell suspension and incubated without shaking at 37 °C for 30 min to separate the aqueous and organic phases. The bacterial cells in aqueous phase were measured at OD_600_ nm and the percentage hydrophobicity (H%) was calculated using the following equation (2):

(2) H% = [(A0 − A)∕A0] × 100

where A0 and A are absorbance values measured pre- and post-xylene extraction.

##### 2.2.1.5 Adhesion to human intestinal epithelial cells

The adhesive ability of *L. paracasei* T0901 to human epithelial intestinal HT-29 cells was test as described by Sornsenee et al. [11]. Briefly, HT-29 cells were grown in Dulbecco's modified Eagle's medium (DMEM) (Thermo Fisher Scientific, Waltham, Massachusetts, U.S.A.) supplemented with 10% fetal bovine serum, 3 mM L-glutamine, and a mixture of antibiotics (50 μg/mL streptomycin–penicillin, 50 μg/mL gentamicin, and 1.25 μg/mL amphotericin B; Thermo Fisher Scientific) at 37 °C in a 5% CO_2_ incubator for 80% confluence. Then, co-cultured with 1 × 10^8^ CFU/mL of *L. paracasei* with intestinal HT-29 cells and further incubated them for 2 h. After incubation, a monolayer of cells was detached using trypsin (2.5%, w/v). The adherent bacterial cells were then recovered by pipetting repeatedly with DMEM and serial dilutions of bacterial cells were then plated on MRS agar. The adhesion ability (%) was calculatedusing the following formula [3]:

(3) % adhesion ability = (V1 × 100)∕V0

where V0 is the initial viable count and V1 is the viable count adhered to the HT-29 cells after incubation.

##### 2.2.1.6 Scanning electron microscopy

The Sornsenee et al. was followed where [11] untreated and treated HT-29 cells with *Lacticaseibacillus* spp. and fixed the control and HT-29 cells treated with *L. paracasei* on a cover slip by 2.5% (v/v) glutaraldehyde (Sigma-Aldrich) for 24 h at 4 °C. Subsequently, the cells were then dehydrated by sequential incubations with gradient ethanol solutions (40%, 60%, 80%, and 95% v/v) for 15 min, each incubation followed by incubation with 100% ethanol (Thermo Fisher Scientific) for 15 min (2 steps). Air dried the cover slips at room temperature (RT) for 30 min, mounted them on stubs, and coated them with gold for 3 min, whereafter visualized the samples under a field emission scanning electron microscope (Oxford Instruments, Quanta, Japan).

#### 2.2.2 Characterization of active antimicrobial substance of probiotic bacterium

##### 2.2.2.1 Pathogenic bacterial cultures

Eight reference strains, including *E. coli* DMST4212, *Listeria monocytogenes* DMST 17303, *Salmonella typhi* DMST 22842, *Salmonella enteritidis* DMST 15676, *Shigella flexneri* DMST 44237, *Staphylococcus aureus subsp. aureus* ATCC 6538, methicillin-resistant *S. aureus* (MRSA), and *Enterococcus faecalis* DMST 4736 were used as pathogenic bacteria [11]. The bacterial strain cultures were incubated on trypticase soy agar (HiMedia) at 37 °C for 24 h under aerobic conditions. Thereafter, cultured the inoculated colonies overnight in brain heart infusion (BHI) medium (HiMedia) at 37 °C. All bacteria were stored at −80 °C in BHI broth with 30% glycerol until testing.

##### 2.2.2.2 Screening of antipathogenic activity

Agar well diffusion assay was used to screen antipathogenic activity of *L. paracasei* cells [11]. Briefly, adjusted the cell density of pathogenic bacteria in BHI medium to 0.5 McFarland and plated them on MRS agar. Cut six wells, each 6 mm in diameter, from the agar, and added 100 μL of 1 × 10^8^ CFU/mL of *L. paracasei* cell suspension to each well. Plates were incubated at 37 °C for 24 h, and measured the inhibition zones.

##### 2.2.2.3 Assessment of antibiotic susceptibility

According to the European Food Safety Authority guideline for microorganism use in food production or additives, antibiotic susceptibility should be tested according to the Clinical Laboratory Standards Institute (CLSI) guidance of 2021 [38, 39]. therefore determined the antibiotic susceptibility of *L. paracasei* cells according to this protocol. Antibiotics (Oxoid, Hampshire, UK) were used for testing in this study, including ampicillin (10 μg), gentamicin (10 μg), erythromycin (15 μg), clindamycin (2 μg), tetracycline (30 μg), and chloramphenicol (30 μg).

#### 2.2.3 Development of banana powder enriched with *L. paracasei* probiotics

##### 2.2.3.1 Probiotic preparation using freeze-dried method

Probiotic bacterial preparations were assembled according to Romyasamit et al. [24], with minor modifications. The *L. paracasei* strain was cultured in MRS broth at 37 °C for 24 h under aerobic conditions. The bacterial cells were then by refrigerated centrifugation (×6000 g, 10 min), washed them three times with sterile DI water, and resuspended them in cryoprotective medium (10% skim milk). Following this, a total of 5 mL cells suspension were frozen in the cryoprotective medium at −80 °C for 5 h and then freeze-dried using a freeze drier (Lyophilization Systems, Inc, U.S.A.) for 24 h at −40 °C to −30 °C, 0.2 mbar. Stored the freeze-dried probiotic powder at 4 °C until further experiments. the cryoprotection of the *L. paracasei* strain were confirmed after FD by scanning electron microscopy (SEM).

##### 2.2.3.2 Cell viability

The viability of freeze-dried *L. paracasei* cells was determined after freezing and during product storages (0, 15, 30, 45, and 60 d) as previously described [24]. Briefly, rehydrated samples in 5 mL of sterile DI. Then plated a total of 100 μL of dilutions on MRS agar and incubated them at 37 °C for 48 h. The enumerated colonies and calculated the survival rate of the *L. paracasei* cells using the following formula [4]:

(4) % survival rate = (N1 × 100)/N0

where N0 is the number of viable cells before freezing (log CFU/mL) and N1 is the number of viable cells after the freezing or freeze-drying storage process (log CFU/mL).

##### 2.2.3.3 Banana powder enriched with probiotics

Formulated ingredients show in Table 1. Thereafter, evaluated this formula using a storage analysis. Firstly, packed the banana powder enriched with probiotics in a matt silver pouch with a zipper, sealed, and incubated at RT for 60 days, whereafter analyzed the water activity (a_w_), moisture content (%), and the number of probiotic bacteria, yeast, and mold every 15 days during storage.

**Table 1:** List of ingredients of probiotic rehydrated beverage.

Total weight (100 g)	
Ingredients	Weight (g)
Banana (Pisang Awak) powder	35
Banana (Lady Finger) powder	15
Granulated sugar derived from nipa palm	30
Non-dairy creamer	9
Skimmed milk	9
Cocoa powder	7
Live *L. paracasei* probiotics	0.01 (containing 1 × 10^7^ CFU/g)

### 2.3 Statistical analyses

All experiments were performed in triplicate. The results showed as the mean ± standard deviation. The data analyzed by one-way analysis of variance (ANOVA) using GraphPad Prism 5 (GraphPad Software, San Diego, CA, U.S.A.). A *p-value* of < 0.05 was considered statistically significant.

## 3 Results

### 3.1 Characterization of probiotic properties

#### 3.1.1 Stimulation of GIT tolerance and enhancement of adhesion ability

**Figure 1:**
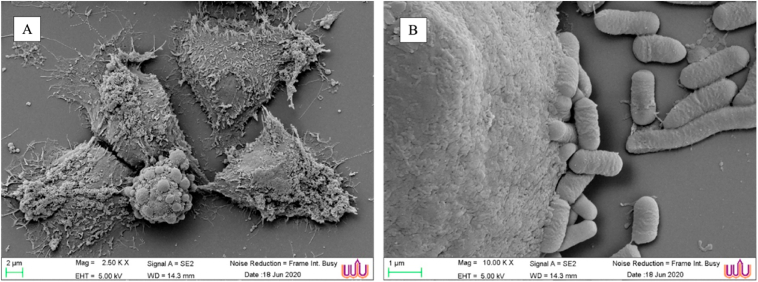
Scanning electron microscope (SEM) analysis of HT-29 cells where the lactobacilli isolates adhere to the surface of HT-29 cells. (A) Untreated HT-29 cells as control. (B) *L. paracasei* adhere to the surface of HT-29 cells.

The analyzed parameters and values to predict the GIT-tolerant properties of *L. paracasei*, as tabulated in Table 2. The *L. paracasei* showed higher acid tolerance and survivability at pH 3 (75.80%) than it did at pH 2 (53%) after 3 h. The *L. paracasei* was tolerant in pancreatic enzyme treatment at pH 8.0 (94.95%) and in pepsin enzyme at pH 2 (53.59%). Moreover, approximately 53% of *L. paracasei* cells survived in 0.3% bile salts. *L. paracasei* showed a high hydrophobicity at 52.38%. It adhered tightly to the HT-29 cells (91.04%). The SEM photograph also revealed a tight interaction between *L. paracasei* cells and HT-29 cells (Figure 1). Therefore, *L. paracasei* cells strongly possessed probiotic properties under stressful GIT conditions, and an enhanced adherent ability to small intestine cells.

**Table 2:** Survival rate (%) of *L. paracasei* cells under physiological parameters in GIT conditions.

Parameters	Survival rate (%)
pH = 2	53.00 ± 4.23
pH = 3	75.80 ± 1.35
Pepsin	53.59 ± 5.45
Pancreatin	94.25 ± 2.96
0.3% Bile salts	53.82 ± 2.95
Hydrophobicity (H%)	52.38 ± 4.26
Adhesion ability (%)	91.04 ± 0.04

#### 3.1.2 Antimicrobial activity

The results revealed that *L. paracasei* cells strongly inhibited *S. aureus* with the highest inhibitory zone, at 21 mm. The moderate inhibitions of *S. enteritidis*, *E. coli*, MRSA, *L. monocytogenes*, *E. faecalis*, *S. typhi*, *S. flexneri* were represented with inhibition zones between 11 mm and 15 mm (Table 3).

**Table 3:** Antipathogenic and antibiotic activities of *L. paracasei* cells.

Antimicrobial activity		
Pathogenic bacteria	Inhibition zone (mm)	
*Escherichia coli* DMST4212	15.33 ± 1.15	++
*Listeria monocytogenes* DMST 17303	14.00 ± 0.00	++
*Salmonella typhi* DMST 22842	11.33 ± 0.58	++
*Salmonella enteritidis* DMST 15676	15.67 ± 0.00	++
*Shigella flexneri* DMST 44237	11.00 ± 0.00	++
*Staphylococcus aureus* subsp. *aureus* ATCC 6538	21.00 ± 0.00	+++
Methicillin-resistant *S. aureus* (MRSA)	15.17 ± 0.29	++
*Enterococcus faecalis* DMST 4736	13.33 ± 0.58	++
**Antibiotic susceptibility**		
**Antibiotics**	**Antibiotic activity**	

Ampicillin **(**10 μg**)**	S	
Gentamicin **(**10 μg**)**	R	
Erythromycin **(**15 μg**)**	S	
Clindamycin **(**2 μg**)**	S	
Tetracycline **(**30 μg**)**	S	
Chloramphenicol **(**30 μg**)**	S	

+, inhibition zone: 6–10 mm, ++ inhibition zone: 11–15 mm, +++, inhibition zone: >16 mm, S = Susceptibility; R = Resistance.

#### 3.1.3 Antibiotic susceptibility

*L. paracasei* probiotics exhibited antibiotic susceptibility (5/6, 83.33%) to ampicillin, erythromycin, clindamycin, tetracycline, and chloramphenicol. However, the strains remained gentamicin resistant (Table 3). The results indicated that *L. paracasei* probiotics strongly exhibited antimicrobial activity against pathogens and high susceptibility to tested antibiotics.

### 3.2 Development of banana powder enriched with *L. paracasei* probiotics

#### 3.2.1 *L. paracasei* probiotic survivability after freeze-dried method

**Figure 2:**
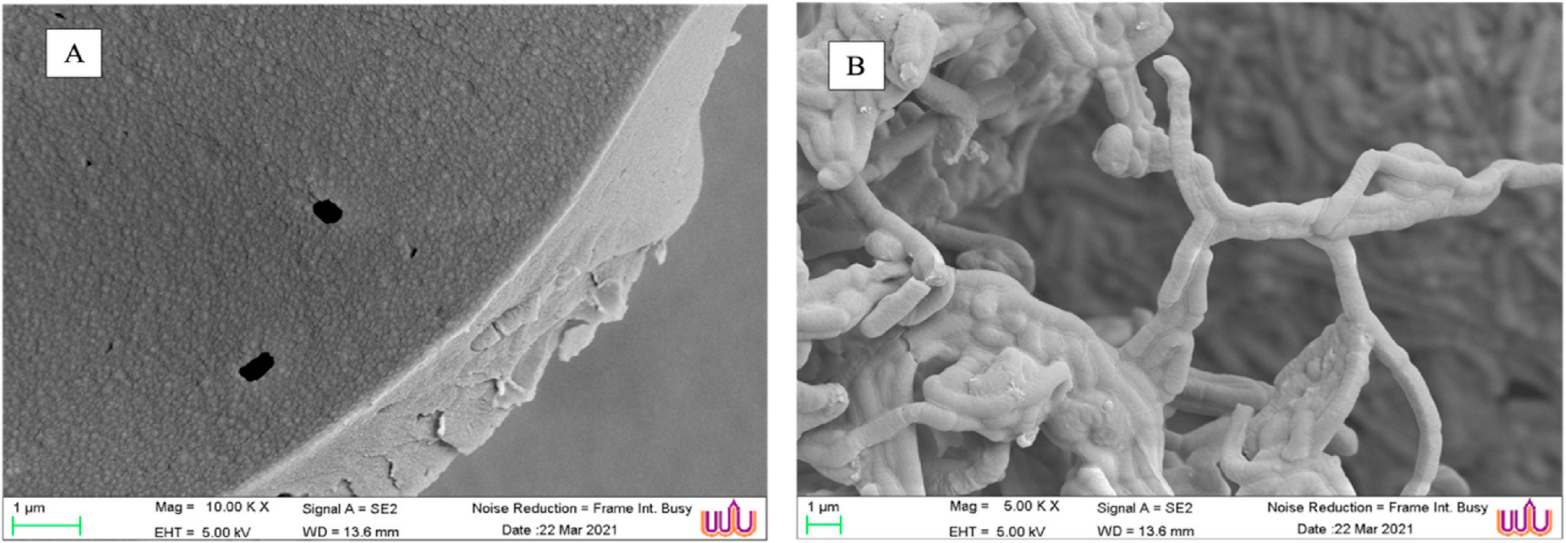
Morphological characterization of *L. paracasei* probiotics incorporated with DI (A) and 10% skim milk (B) after freeze-dried method.

Cell viabilities of *L. paracasei* before and after the freeze-dried methods were not significant. The number of bacteria was 11.04 ± 0.12 and 11.01 ± 0.09 log CFU/g, respectively. The morphological characterization of the *L. paracasei* cells and skim milk cryoprotectant was determined using SEM analysis, to ensure that FD could maintain the sustained probiotics, as shown in Figure 2.

#### 3.2.2 Banana powder enriched with probiotics

The product quality of the freeze-dried *L. paracasei* probiotics supplemented with banana powder beverage during storage for 60 days at RT showed in Table 4. This result showed that the a_w_ (0.235–0.363) and percentage of moisture content (2.75%–3.80%) increased in time-dependent manner. The number of probiotic cells decreased from 8.01 to 7.10 log CFU/g in a time-dependent manner. Nevertheless, the cell decreases were gradual, and reached concentrations close to the initial concentrations of probiotics added (log 7 CFU/g). Non-yeast and mold cells were detected. Therefore, the data suggested freeze-dried *L. paracasei* probiotics were stable. The probiotic powder product also remained safe after prolonged storage.

**Table 4:** Water activity and microbiological analysis of the probiotic powder product at RT for 60 days.

Days	Water activity (a_w_)	Moisture content (%)	Probiotics (Log CFU/g)	Yeasts and molds (Log CFU/g)
0	0.235 ± 0.00^a^	2.75 ± 0.05^a^	8.01 ± 0.09^ab^	ND
15	0.253 ± 0.00^b^	2.94 ± 0.03^b^	7.96 ± 0.10^cd^	ND
30	0.295 ± 0.00^c^	3.13 ± 0.02^c^	7.90 ± 0.18^ef^	ND
45	0.323 ± 0.00^d^	3.49 ± 0.06^d^	7.33 ± 0.35^ace^	ND
60	0.363 ± 0.00^e^	3.80 ± 0.02^e^	7.10 ± 0.17^bdf^	ND

Values are mean ± SD; ND = not detected; a–f values are significant differences between storage at different times by one-way ANOVA and the multiple Bonferroni test (*p* < 0.05).

## 4 Discussion

Probiotics must physiologically survive GIT conditions such as low pH, bile salts, pepsin, pancreatin, and hydrophobicity, and possess adherent ability to small intestinal cells [40]. *L. paracasei* cells tolerated these GIT stresses, affirming the results of our report [11]. The cells remained viable at low pH. The acid tolerance was in accordance with previous reports [41, 42]. Also, 50% of *Lacticaseibacillus sp*. cell viability was acceptable as a potential probiotic at pH 2–3 for 2–3 h [41,43]. The probiotic cells reduced at pH 2, which represented the acidity in stimulated gastric juice. However, this phenomenon is rare in the GIT, excepting during fasting [41]. The *L. paracasei* probiotics appeared to survive better with a food carrier, at approximately pH 3.0–5.0 in the GIT. There was no significant effect on the survivability of *L. paracasei* cells (95%) in alkaline condition (pH 8.0) in stimulated small intestinal juice. The *Lacticaseibacillus* cells maintained themselves in a wide range of pH values. This could be an adaptation to environmental stress through various physiological and biochemical changes, including cell membrane modification, activated H^+^-ATPase activity, and elevated alkaline homeostasis in the cytoplasm by the production of several metabolic enzymes and stress proteins [40, 44]. Moreover, bile salts represented some conditions of the small intestine environment. The intestinal bile concentration is 0.3% and maintains food in the small intestine for 4 h [45]. The *L. paracasei* cells were stable to survival at 4 h, in accordance with previous studies [14, 43]. Cell surface hydrophobicity were to indirectly estimate the adherence ability to epithelial cells [45]. In addition to the high auto-aggregation of the *L. paracasei* cells (∼70% of viability) demonstrated by the Sornsenee et al. [11] study, our probiotic cells showed high cell surface hydrophobic affinity and adhesion ability. Indeed, the results indicated that the *L. paracasei* cells represented a promising probiotic candidate for further use in functional food application. They could notably survive in the acidic stomach environment and strongly reached the areas of beneficial activity in the small intestine and colon.

Most probiotics claim to have the status of “generally regarded as safe” (GRAS) [1, 2]. The safety of the original *L. paracasei* probiotic was clearly seen because it was isolated from edible fermented palm sap [11]. Furthermore, to allay fears surrounding antibiotic resistance concerns for the safety of probiotic use for human consumption [46], Moreover, non-antibiotic resistance to the selected antibiotics by *L. paracasei* probiotics.

Functional properties of probiotics were critically determined against pathogens for an alternative antimicrobial therapy [47]. Cell supernatant of *L. paracasei* species exhibited high activity against several enteric bacteria, particularly, against *S. aureus*. The antipathogenic property of *L. paracasei* in our study was similar to that of several previous studies [47, 48, 49, 50]. Furthermore, Sornsenee et al. reported that antipathogenic activity of the selected *Lactobacillus sp.* could produce proteinaceous agents and bile salt hydrolase activity to inhibit spore germination of some pathogens [11]. The results revealed that *Lactobacillus* strains isolated from curd and fermented durian (Tempoyak) samples produced some effective antimicrobial substances such as organic acid, exopolysaccharide, and bacteriocin. [50, 51]. Commercially available probiotic drinks containing *L. casei* DN-114001 reduced antibiotic-associated diarrhea in German patients through the production of many bioactive metabolites, which conferred host benefits when consumed [52].

In this study, the incorporation of skim milk in the freeze-drying process could have assisted in maintaining *L. paracasei* cell survival (∼10^11^ CFU/g). SMP helps to protect probiotic cells by adsorption on the cell surface to form as a viscous layer, causing partial efflux of water from the cell, inhibiting ice crystal growth, and maintaining ice amorphous structure in close cell proximity [22]. The technical application of functional probiotic food to benefit GIT purposes. After 2 months storage, the *L. paracasei* probiotics maintained high survivability at initial addition (log 7.10 CFU/g). To ensure health benefits by addition of probiotics, probiotic cells must be present in the product at minimum 10^8^ CFU/g or 10^6^–10^9^ CFU/g (daily intake) [21, 53]. This recommendation is in accordance with our range of probiotic findings after continuous freeze-dried probiotic storage. Moreover, our finding showed that the SMP-lyophilized probiotic cell survival was similar to those of *Lacticaseibacillus* strains that were microencapsulated with whey protein isolate and fructo-oligosaccharides over 30 days of storage at 4 °C [21]. The average cell survival for *L. casei* is ≥ 7.61 log CFU/g [21]. In long-term storage, our finding also showed undetectable microorganisms (mold, yeast, and pathogenic bacteria) because the a_w_ (0.235–0.363) and moisture content (2.7%–3.8%) were low, thus inhibiting their growth, spore germination, and toxin production [33, 34]. Good rehydrated-products should present lower a_w_ (<0.6) and moisture content (<25%) [33, 34]. The a_w_ (0.235–0.363) and moisture content (2.7%–3.8%) were increased in time-dependently during storage. While probiotic cells were decreased by time of storage (log 8.01–log7.10 CFU/g), the reduced probiotic survivals were correlated with higher a_w_ and moisture content [34]. In accordance with the study of Savedboworn et al. (2020), for example, the a_w_ increase in vacuum-dried *L. casei* TISTR 1463 with plant protein protectant ranged from 0.304 at the initial storage time to 0.521 at the end storage time [54]. The study also suggested that the reduction of vacuum-dried probiotic viability corresponded to the increase of a_w_. [54]. Tymczyszyn et al. suggested that the critical a_w_ value of 0.7 induced membrane damage in vacuum-dried *L. bulgaricus* CIDCA 333 [55]. Furthermore, the proper packaging and temperature are suggested to be associated with the a_w_ and moisture content accumulation [34]. However, the increase in a_w_ and moisture content values in this study still fall within acceptable ranges of a rehydrated product. Control of a_w_ correlating with moisture content in a dry product also maintains proper non-microbial effects of product properties—such as structure, texture, and density [33, 34].

## 5 Conclusions

From the results, *L. paracasei* T0901 isolates have beneficial properties including stimulated GIT host benefit and antimicrobial activity. This is the first report on the survival of *L. paracasei* in banana powder and the development of banana powder enriched with *L. paracasei* probiotic products. During storage for 60 days at RT, the final product contained a sufficiently high level of probiotic cells (7.10 log CFU/g). In addition, the final product had a low aw (∼0.363), indicating a good keeping quality. Thus, this product may be used as a functional food and nutritional product.

## Declarations

### Author contribution statement

Phoomjai Sornsenee: Conceived and designed the experiments; Performed the experiments; Analyzed and interpreted the data; Wrote the paper.

Siriphorn Chimplee: Performed the experiments; Analyzed and interpreted the data; Wrote the paper.

Phanvasri Saengsuwan: Performed the experiments; Contributed reagents, materials, analysis tools or data.

Chonticha Romyasamit: Conceived and designed the experiments; Performed the experiments; Analyzed and interpreted the data; Contributed reagents, materials, analysis tools or data; Wrote the paper.

### Funding statement

This research did not receive any specific grant from funding agencies in the public, commercial, or not-for-profit sectors.

### Data availability statement

Data included in article/supp. material/referenced in article.

### Declaration of interest’s statement

The authors declare no conflict of interest.

### Additional information

No additional information is available for this paper.
